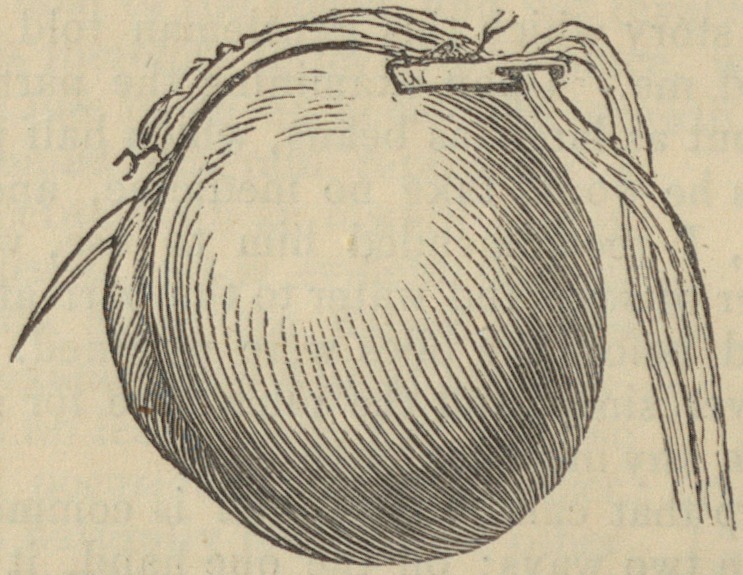# Observations on Injuries and Diseases of the Rectum

**Published:** 1833-10

**Authors:** 


*Observations on Injuries and Diseases of the Rectum.* By HERBERT MAYO, F.R.S., Surgeon to the Middlesex Hospital. London, 1833. 8vo. pp. 220.

WE have often thought that the most agreeable moment in a professional man's career is that in which he receives the appointment of physician or surgeon to a metropolitan hospital. Opportunities of boundless improvement are placed before him, and a thousand cases of undefined disease await but the approach of a master-mind to show that their phenomena are as regular as those which physiology presents, and that what is called an anomaly in nature is merely a law not understood. When compared with the occasions of undisturbed study ever offered to the hospital practitioner, all others appear like hasty snatches of instruction, tolerated indeed, but not encouraged; and, should the healing art ever rise to the dignity of a science, it will be to these fortunate students of nature that it will be indebted for its elevation. Whoever therefore is invested with this important charge, should be considered as pledged to the advancement of the art which he professes; and we cannot be thought unreasonable in requiring that a man who has had a myriad of important cases under his care should teach us the diagnosis of some obscure disease, or the method of palliating the effects of some incurable disorganization; that he should either clear up some great truth, or at least pave the way for it, by exploding some long-established error. This pledge Mr. Mayo has redeemed; and, though it should be objected that so short a treatise is rather the outline of a book than a book, it must be allowed that every page gives ample promise of the most finished excellence.

In the first chapter, "on Laceration of the Rectum," our author remarks, that constipation is a common cause of this accident: this is true; but he might also have added, that the purging and straining which attend on the operation of drastic medicines, or which may even arise without any very obvious reason, will occasionally produce the same effect. The dose of calomel, scammony, and gamboge, for example, with which Madame Noufer was wont to assist the operation of her anthelmintic, might easily have lacerated the rectum in a predisposed subject.

In the first case given by Mr. Mayo (p. 3) he says, "Upon examining the bowel, I found a small transverse fissure in the lining membrane, at the back part of the bowel immediately within the sphincter, *which is the point where fissure is most common.*" The expression which we have marked in italics is perfectly correct; but the reader must not suppose that fissure is confined to this point, though most common there. A case was recently communicated to us by an eminent surgeon, in which the laceration occurred at the fore part, opposite the end of the os coccygis.

A complete rupture of the rectum into the vagina, occurring independently of parturition, must be considered as one of the rarest of accidents: the following, however, is an example of it.

"IV. ******, ætat. forty, naturally of a very constipated habit of body, and at the time being on a journey, on striving to relieve the bowels, which had not acted for many hours, felt something give way, (to use her own expression,) and on the following morning some fæces passed per vaginam. On examination by the vagina and rectum, a transverse rent was found two inches within the parts, sufficiently large to admit the end of the finger. The only treatment adopted in this case consisted in frequently and carefully cleansing the part by injections of water, and regulating the state of the bowels by proper medicines. The patient entirely recovered. In five weeks the fæces had ceased to pass per vaginam." (P. 13.)

It is highly probable that in this case a slight fissure existed previous to the straining at stool, and was aggravated by this exertion into a considerable rent. This dreadful accident, however, is most frequently the result of unfavourable parturition, and is remarkably well discussed by our author in the following passage:

"But the most frequent instances of laceration of the rectum into the vagina result from other causes than those already described, and are produced by violence acting from the vagina towards the bowel. This violence is the pressure of the head of the child in labour. The degrees of injury which it occasions are very various.

"Occasionally the injury is the same in its degree as in Case IV. being a laceration within the sphincter of trifling extent, which heals as readily when ensuing upon this cause as where it follows any other.

"Frequently the laceration is limited to the perineum, is quite external, and involves at the utmost the marginal fibres of the sphincter muscle. In this instance again, the recovery is certain and spontaneous: but it is necessary in both instances to employ cleanliness and rest to promote the reparation of the part.

"There are however severer cases, in which the sphincter being completely ruptured, the extremity of the bowel communicates with the vagina by a longitudinal fissure from three-fourths of an inch to an inch in length. It is known in such a case that all the fibres of the sphincter are torn through, by the total want of tonic contraction of the bowel at the part where it begins to be entire.

"In many instances in which this accident happens, spontaneous reparation of the part does not take place. The force of the sphincter muscle is employed in keeping the rent wide open; and the fæces continually passing through it contribute to prevent its uniting.

"In a conversation with Mr. Copeland, I learnt, that in a case of this description he had successfully employed the following method. It occurred to him, that if the sphincter were divided at a second part, its strain upon the rent into the vagina would be greatly lessened, and that the chance of reparation would be proportionately increased. I understood from him, that he had divided with this object the sphincter laterally, and that the case had turned out completely to his wish.

"Soon after this conversation, a similar case came under my own care.

"VI. I was requested by a medical practitioner to see, in consultation with him, Mrs. Quye, who had been confined of her fifth child eight days before, March 31, 1830. The labour had been rapid, and the pressure of the head of the child had ruptured the perineum and the sphincter. The fæces passed freely through the vagina by a gaping fissure nearly an inch in length. As the edges of the fissure were not cicatrized, I thought the present a very favorable opportunity for repeating Mr. Copeland's operation. To give the parts every chance, I divided the sphincter muscle upon both sides, performing therefore on either side the operation for fistula ani. A small strip of lint was introduced into each wound. The edges of the original rent were afterwards washed daily with a solution of nitrate of silver, and fresh lint was replaced in the incisions as often as it was removed by the passage of fæces. The original rent healed very speedily: when it was nearly closed, the lateral wounds were allowed to unite. In five weeks from the operation the incisions had healed, and the patient had recovered the use of the sphincter. She has continued perfectly well to the present time, and was safely confined of another child in November 1832.

"The remarkable success which has attended this practice in recent cases has induced me to determine to employ it, after the manner recommended by Dieffenbach, in cases in which laceration of the sphincter has occurred, and has not been remedied at the time.

"In cases of long standing, it is however obvious that the lateral division of the sphincter is a part only of the operation requisite for the restoration of the parts. It is necessary besides, at the least, to produce a granulating surface upon the edges of the fissure.

"And as the parts in this class of cases have lost, from time, the tendency which exists in the freshly-torn parts to come together, I conclude that it is absolutely necessary to use ligatures to bring them and to hold them in contact. In other words, I recommend the performance of the following operation. The first step of the operation consists in paring the edges of the fissure; the second is the introduction of sutures, to be tied afterwards in the vagina; the third is the division of the sphincter on either side; the operation is completed by tying the ligatures in the vagina, and by introducing a strip of lint into each of the two lateral sections of the sphincter. I am the more sanguine as to the general result of this operation, since I had the good fortune to apply ligatures successfully to an old laceration of the rectum into the vagina, without the assistance of the method which I have now described, and therefore under circumstances considerably less favourable. Much, after all, in such an operation, depends upon causes over which the surgeon has a very imperfect control. Inflammation of the parts supervening, or diarrhœa, would still be liable to render the operation ineffectual. But the surgeon of course defers attempting it, till the patient is in her best state of health, and the bowels have been thoroughly unloaded by repeated doses of opening medicine. The bowels should not be moved for several days after the operation." (P. 21.)

We will freely confess, however, that, in our opinion, cases like that of Mrs. Quye are among the rarest in the annals of obstetric surgery, and that, for one woman who suffers this laceration through the labour-pains, a thousand owe it to the forceps.

The second chapter, "Of Protrusion of the Rectum,"

treats of the disorders ordinarily called Prolapsus or Procidentia ani, and is replete with sensible and practical remarks. Dupuytren and others "have described prolapsus ani as consisting in an extrusion of the mucous and submucous coats alone, through the action of the muscular coat of the bowel." But Mr. Mayo corrects this error, or, at any rate, shows that Dupuytren's theory is not universally true, by giving a woodcut of a preparation in King's College museum, in which it is clearly seen that the muscular layer is inverted as well as the lining membranes. Mr. M. has given the diagnosis of prolapsus ani from piles in a very satisfactory manner: indeed, it is surprising to us that these affections should be confounded; for, though no doubt there are piles which, when quiescent, are only folds of membrane, still these troublesome tumors cannot have the intestinal channel opening in their centre, as a prolapsed rectum would, and consequently cannot be mistaken for it by any one possessing an ordinary share of surgical tact.

In the third chapter our author treats "of Bleeding from, and Pain in, the Rectum." Hemorrhage from this part is to be viewed "as a warning that there is something wrong in the habits of life; that the diet is too stimulating; or that sufficient exercise is not taken; or that the secretions from the bowels are not sufficient in quantity. The recurrence of the attack may probably be prevented by attending to the precautions suggested by the preceding views. The attack itself may be relieved by the use of gentle aperient medicines, with cold bathing to the part." (P. 50.)

If the discharge has continued a long time, however, astringent enemata must be used.

We regret that Mr. Mayo has not touched upon the interesting subject of arterial hemorrhage from the rectum. In these cases the patient imagines himself to be suffering merely from bleeding piles, and the disease is only detected by an examination, when an artery is discovered bleeding *per saltum* at the apex of a minute fungous tumor. Dr. Abercrombie has given a good account of this disease, and observes, that the cure consists in tying the artery, and, if that should not be sufficient, the spongy mass is to be freely touched with lunar caustic.

In the next chapter we come to the consideration of the most common disease to which sedentary man is subject—the Piles. We like Mr. Mayo's definition of these tumors very much.

"Piles, or hemorrhoïds, are soft tumors, which form either within the rectum or about the anus. In the first case they are covered with the mucous membrane of the intestine, and are termed inward piles. In the second case they are covered entirely, or in part, with the common integument, and are termed outward piles." (P. 58.)

In the first section the subject of inward piles is so well discussed, that we cannot resist the temptation of laying a considerable portion of Mr. M.'s observations before our readers.

"SECTION I. *Of Inward Piles.* Inward piles vary from the size of a pea to that of a large walnut. They are sometimes single, at other times there are several. Sometimes they grow immediately within the sphincter; at other times at some distance above it. They are sometimes attached by a narrow pedicle; at other times they have a broad or elongated base. In some cases they do not protrude beyond the sphincter; in others, they are extruded at every motion.

"A pile protruding at each action of the bowels, and afterwards returned by pressure, in what does it differ from a prolapsus? It differs in this respect essentially: it is a tumor formed internally to the muscular coat of the bowel, and not involving it. The canal of the rectum is therefore in its natural place, without elongation or eversion, the pile being an accidental growth of its inner surface. The adjoined diagram represents a section of the rectum with an inward pile in a state of protrusion. It is evident that, if there be a doubt as to the nature of the protrusion, an examination will at once remove it. It is no less evident that the two complaints will occasionally exist together. The one indeed naturally leads to the other. Neglected piles often prove a source of irritation sufficient to produce prolapsus, which ceases to recur upon the removal of the cause which occasioned it.

"The colour of internal piles varies with their condition. It is sometimes that of the bowel itself, a shade of reddish brown; at other times a dark purple, approaching to black; at other times a bright red. Internal piles are particularly liable to bleed; yet in some instances they exist for several years without bleeding.

"The following case will serve to exemplify one form of this disease, as well as the efficacy of a very simple remedy, attention to which is in every case of the greatest advantage.

"A gentleman, ætat. fifty-seven, of a spare frame of body, and of temperate habits, consulted me for piles. Thirty years before, when in good health, he lost by stool a large quantity of blood; in a few hours the anus became tumefied, knotty, painful. The following day he was obliged to ride thirty miles on horseback. As he proceeded on his journey, he became better, and on the ensuing day he felt quite well. About four months subsequently he had an attack of the following description: The anus, without any assignable cause, became tender, tumefied, and painful. This state of things continued three days; on the third night he became better; some discharge of mucus, with blood, took place, and in a day or two he was well again. These attacks were repeated during the following twenty years, and usually recurred once in three months: they were extremely severe and distressing. This gentleman, who is in the medical profession, entertained a strong aversion to medical or surgical treatment: he therefore bore the pain, and contented himself with bathing the parts with cold water.

"During the last ten years the character of the complaint had been different. The patient had suffered less severely than before, but he had suffered constantly. The bowels had acted regularly, and that without pain; but every afternoon, about one o'clock, the part had become heated and uneasy, indisposing him to exertion of any kind. Towards evening the uneasy sensations had left him.

"Such was the story which this gentleman told me a year ago, when he consulted me. Upon examining the part, I found two internal piles, about as large as beans, which half protruded upon his straining. As he would take no medicine, and use no medicated application, I recommended him to use, with scrupulous regularity, a lather of soap and water to the part after each action of the bowels, and before the piles were returned. This practice he has followed ever since, and the piles have for several months ceased to give him any inconvenience.

"The best soap that can be employed is common yellow soap. It is serviceable in two ways: on the one hand, it removes completely any remains of fæcal matter; on the other, it acts as an astringent. In the case which I have described, the latter object was, I have no doubt, quite as important as the former; the piles being in that simply uneasy state, not very irritable and angry, in which astringent applications are commonly found useful. But the first object is likewise one of great consequence. The want of complete cleansing of the bowel is one of the causes which most tend to the production of piles, whether external or internal. Water alone is not sufficient to cleanse the part; complete ablution with soap as well is necessary for this purpose. Those who are thus scrupulously cleanly suffer less from piles than other people.

"A remedy very commonly tried for indolent internal piles, and which in many cases proves of service, is the Confectio piperis composita of the Pharmacopœia. This remedy is to be taken internally, in the dose of a drachm, two or three times a-day; it seems to act as an astringent when applied locally, giving a salutary tone to the vessels of the part." (P. 60.)

Our author sees no danger in removing piles by the ligature, provided the following precautions are attended to:

"The patient must not undergo this trifling operation when labouring under any casual indisposition.

"Before its performance, the bowels should have been several times freely moved with aperient medicine.

"If the patient should be unable easily to extrude the tumors, they may be brought to protrude upon the patient sitting over hot water, and endeavouring to force them down, or after the use of a lavement of warm water.

"The ligature should be drawn round the base of each tumor so tightly as thoroughly to strangulate it. To ensure this object it is desirable, after the ligature is applied, before finally tying it, to cut into the pile; after which precaution the ligature may be drawn much closer than it would otherwise be possible. If the pile be of large size, it is desirable, for the same purpose, to pass a double ligature through the tumor with a needle, and then to tie either half separately, in the manner shown in the following diagram.

"If there are several internal piles, it is necessary that all should be tied.

"After tying a pile, the ligature is to be cut short, and the ends are to be returned with the strangulated pile into the rectum. If much pain follow the operation, it may be allayed by a dose of laudanum. The pain generally in a short time subsides entirely; and it is only requisite for the patient to remain at rest for the next few days, when the ligatures and the piles come away without his knowledge. But occasionally fresh pain supervenes on the second or third night. When this happens, it is presumable that the pile has not been entirely strangulated: the parts should then be examined, and the ligature should either be removed for the time or drawn closer, according to the state of the parts. If much pain supervene after tying piles, it is always safe and useful to apply leeches to the extremity of the bowel." (P. 71.)

In the next section the subject of outward piles is ably discussed; and a curious instance is given, showing the possibility of mistaking an external for an internal pile. A physician, whom Mr. Mayo had formerly attended for inward piles, came to town to consult him again. On his arrival in London, and before he was seen by our author, the part was examined by an experienced surgeon, who told him that the tumour might be returned within the sphincter, but that the necessary pressure would give considerable pain. The following passage will show the aspect of the case, while it exhibits Mr. Mayo in a very favourable light, as a thinking and analytical practitioner.

"The appearance which the part presented was that of a solid tumour on one side of the anus, extremely firm, partly covered with tense and shining integument, partly with the mucous membrane of the margin of the bowel. On examining the rectum, the swelling and hardness were found to extend an inch within it. It was evident that no operation would be of service; and that as the tenderness and pain in the part, though still considerable, were progressively lessening, no treatment would be necessary beyond the use of a poultice and occasional doses of opening medicine, with abstinence from wine and heating food. The tumour I concluded to be an outward pile, no part of which would on its diminution be drawn or forced within the sphincter. The result proved that this opinion was right: the tumour only shrunk.

"The case which has been described appeared to me interesting in three points of view.

"In the first place, it was a striking instance of the possibility of mistaking an external for an internal pile. This mistake might have been of consequence; if it had been acted on, the patient would have been put to great pain, and the complaint, instead of being benefited, would have been materially aggravated.

"In the second place, this case established that an hemorrhoidal tumour may form in the part of the bowel surrounded by the sphincter. The swelling was not merely prominent by the side of the anus, but could be traced some way within the sphincter. I mark this circumstance, because I believe that it is laid down on no common authority, that the pressure of the sphincter precludes the formation of the hemorrhoidal tumor within its circumference.

"Thirdly, the preceding case gave me an opportunity of ascertaining what becomes of inward piles, when they cease to give uneasiness and to be felt by the patient. This gentleman had consulted me two years before for an inward pile, which protruded on the action of the bowel, as a round and vascular and turgid knot. By the use of appropriate remedies he had entirely recovered; but I found, upon examining the bowel on the present occasion, a soft insensible pendulous process within the rectum, nearly cylindrical, about an inch in length and a third of an inch in diameter. This had been the inward pile with which he had formerly suffered; it had shrunk, and little remained but the elongated membranes which had formed its covering." (P. 86.)

The subject of the fifth chapter is Fistula Ani. Our author is singularly happy in giving the definition and brief history of a disease; as in the passage we are about to quote.

"By a fistula is meant a narrow channel or sinus, secreting purulent or serous discharge, and having an external opening near the anus, through which the matter has vent. The opening of a fistula is often extremely small, so that there may be difficulty in finding it. The channel itself, or fistula, is usually a little larger than a common probe: it is sometimes strait, sometimes crooked: its length may vary from half an inch to several inches. Towards its inner extremity a fistula reaches the coats of the rectum: it may terminate inwards by a small aperture of communication with the intestine, or blindly, as a cul de sac. There may be one fistula, or there may be several; and in the latter case the fistulæ may or may not reciprocally communicate.

"A fistula is a consequence of an abscess, which, when it has broken or been punctured, contracts to such a narrow channel as has been described, which continues permanent. The complaint requires to be studied separately in its two stages, first as an abscess, secondly as a permanent sinus.

"Abscesses near the rectum again admit of a practical distinction into two kinds; either they are small and superficial, which is the character of those that lead to fistula; or they are deep-seated, when they often contain large accumulations of matter, but rarely produce the secondary complaint." (P. 101.)

Mr. Mayo has forgotten to notice the frequency of this affection among tailors, which forms an exception to his rule, that diseases of the rectum are more common among the higher than the lower classes of society. Mr. Thackrah, in his work on the diseases of artisans, informs us that a fistula club exists among the journeymen employed by Stultz; so that sartorial prudence has suggested the necessity of insuring themselves against the loss of wages entailed on them by this distressing complaint: it were well, however, if the prophylaxis proposed by this benevolent surgeon could be adopted, and the attitude of the workmen made more compatible with health.

The following case illustrates a cognate subject:

"William Knight, æt. sixty-five, was admitted into the Middlesex Hospital, August 9, 1832. For five months previously, he had experienced violent aching pains about the hips and loins, and down the back of the thighs to the knees, slight dysuria, and habitual constipation of the bowels. During the last six weeks he had suffered more acute pain within the anus, shooting to the projections of the ischia and round the haunch bones. He passed urine with great difficulty, and could scarcely void it unless at the same time he strove to empty the bowels. Upon examining the rectum, I found a collection of fluid in the region of the prostate gland. This patient experienced relief from the use of the hip-bath, with an opiate suppository at night, and mild aperient medicines. But in five days after his admission, the abscess broke into the rectum, discharging as he thought a pint of matter, which was followed by the complete removal of all his symptoms, and a very speedy recovery. He left the hospital perfectly well on the 28th of September." (P. 110.)

The sixth chapter treats of "Constipation of the Lower Bowels, and of the Use of Instruments." Mr. Mayo observes, and the remark is an acute one, that constipation may occur from want of fæces; and he gives two cases which exemplify, in different degrees of intensity, "the consequences which result when the blood is not relieved of this excretion." Our author might have noticed the analogy between this disease and Ischuria renalis; and, as this rare anomaly usually terminates in serous effusion on the brain, so in the following curious case, narrated by Mr. M., obstinate constipation was found to be connected with *ramollissement* of the spinal cord: it would be difficult, we think, to decide which was cause and which effect. Our author supposes the primary disease to have been in the cord.

"I was consulted in the case of a young lady, one of whose symptoms was obstinate constipation of the bowels, requiring that she should take nightly from twenty to thirty grains of compound extract of colocynth, to produce an action of the bowels the following day. She had been ill four years, and her sufferings had commenced with severe pain across the belly, and obstinate costiveness. After a fortnight's illness the constipation yielded; but one leg became feeble, and the knee of that side was frequently spasmodically bent. This complication of palsy and spasm soon after affected the opposite leg; afterwards one hand became feeble and contracted. These symptoms grew upon her; but she retained a remarkably fine complexion, and had the appearance, when making no exertion, of perfect health. I entertained little doubt that all the symptoms in this case originated in an affection of the spinal marrow. The vertebral column was indeed perfectly strait and even; but the patient often experienced pain at the lower part of the dorsal portion, and pressure there gave her uneasiness. I recommended that issues should be made at the lower part of the back. The remedy was followed by great relief of all her symptoms. The legs seemed less weak, the knees were not so frequently or so painfully contracted, and the bowels acted with half the usual dose of drastic purgatives. This improvement however was temporary only; and, disappointed of obtaining permanent relief, this patient consulted other surgeons, as she had consulted several before she applied to myself. She died six months afterwards; and, on examining the spinal cord, it was found for the length of two inches in a state of softening at its lumbar portion." (P. 141.)

The chapter concludes with some observations on the mischief which may be caused by the hasty or injudicious introduction of instruments into the rectum. These cautions are now more necessary than formerly, as within a few years enemata seem to have become fashionable; and the violent encomiums lavished on these inelegant purges have induced many unprofessional persons to provide themselves with a clyster-pipe, as if it were an article of prime necessity in every decent household.

"Stricture of the Rectum" is the subject of the seventh chapter. Mr. Mayo very justly observes, that "the rectum resembles the œsophagus in its affinities for disease, and stricture is as rare in it as ulceration and schirrus are common." (P. 153.) In detailing the symptoms of this morbid affection, our author mentions flattened fæces as one of them. Of course we do not object to this; but we could have wished that he had added the fact, that flattened fæces may and do occur without stricture of the rectum. Generally speaking, Mr. M. thinks the use of bougies sufficient, and is averse to the division of the stricture by the knife.

In the last chapter, which treats of "Cancer of the Rectum," we find three operations recommended as likely to afford some relief in this formidable disease. The first is the division of the scirrhus:

"Instances of fungoïd scirrhus occasionally present themselves, in which the quantity of the malignant growth is so considerable, and the sensibility of the part so great, that the bougie cannot be introduced or borne. When this is the case, the channel may be enlarged by the division of the scirrhus. No ill consequence follows the operation, and great relief is obtained by it. Of course this practice is only applicable when the part to be divided is within reach of the finger.

"Mary Woolgrove, ætat. thirty-two, was recently admitted into the Middlesex Hospital. In the year 1818 she had been cut for fistula, and since that time had never been entirely free from occasional discharges of blood and mucus from the bowel. But it was not till three years and a half ago, that pain and obstruction, and other symptoms of carcinoma, appeared. At the period of her admission she was greatly extenuated, having suffered for several weeks constant painful purging of liquid matter. The anus was indurated, and surrounded with scirrhous nodules partly in a state of ulceration. Upon an examination of the rectum, the finger was stopped at an inch within the gut by a mass of fungoïd scirrhus, through which an urethra-bougie could only be passed. By means of opiates the pain which this patient suffered was mitigated, and the purging checked: I then tried to enlarge the passage by the use of bougies. But the attempt was ineffectual, and violent liquid purging returned. Under these circumstances I determined to divide the scirrhus. For this purpose I introduced the blade of a strong strait probe-pointed bistoury upon the fore-finger of the left hand, and divided the scirrhus towards the sacrum, gaining space enough to allow the finger to be passed further into the bowel. I then divided in the same manner the part beyond. The scirrhus terminated, as I had anticipated, at three inches within the anus, so that the operation was entirely successful. It has given the patient great relief, who now has a free passage through the part, which is besides less sore and painful than before." (P. 204.)

The second is the excision of the carcinoma; and a case is given, in which Mr. Crosse, of Norwich, performed this operation, with temporary benefit to the patient, who was even able to resume his occupation as a hackney-coachman for a short time, but sank under a return of the disease in a few months. "But," says Mr. Mayo,

"We may go further, and inquire whether a part of the entire cylinder of the bowel may not be removed along with the scirrhus, the matrix of the disease with the disease itself.

"M. Lisfranc recommends, and has several times performed, excision of the lower part of the rectum, in cases in which the finger can be passed completely beyond the limits of the disease, and the intestine at three or three and a half inches from the anus is ascertained to be healthy.

"I performed this operation in the case of a woman about forty years of age, in whom the inner surface of the bowel began to be ulcerated half an inch within the orifice. The ulcer extended round the rectum, and was upwards of an inch in breadth: there was considerable induration. The patient had suffered long and severely, and could not quit the recumbent posture.

"The steps of the operation were, first, an oval incision through the skin around the anus, at a distance of half an inch from the mucous membrane; secondly, dissection of the bowel from the adjacent parts, and securing the vessels as they were cut through; thirdly, division of the bowel, by which the already isolated part including the disease was separated from the sound bowel above.

"The results of the operation were as follows. The patient expressed a strong sense of relief and comfort almost immediately after it was concluded. She felt, to use her own expressions, that the cause of her previous sufferings was gone. In a month her appearance became surprisingly altered. The extenuation and distress of countenance that had before been so remarkable left her, and she became a fat and cheerful and comely person. I was now apprehensive of one of two alternatives, either that the hollow cylindrical cicatrix leading to the bowel would contract and form a troublesome stricture, or that, as the sphincter was completely removed, there would be distressing incontinence of fæces. Neither of these evils, however, occurred. The cicatrized surface did not contract; and, unless the bowels were in a very loose state, the patient was always aware when their action was likely to take place. But a serious evil ensued, which I had not anticipated, and could not obviate. Prolapsus of the bowel came on; some length of intestine was gradually pushed out in a state of eversion; and the mucous surface, irritated by exposure and pressure, became a new and constant source of uneasiness.

"About two years after the operation this patient died of an attack of abdominal inflammation. The mucous membrane adjoining the cicatrix had begun anew to ulcerate." (P. 211.)

The large extracts we have made from Mr. Mayo's treatise will show our opinion of its excellence; but, considering the nature of the subject, there is one feature which deserves an especial panegyric: it is not addressed to hypochondriacal patients, but to scientific surgeons,—to those, in short, who are best qualified to appreciate its merits, and supply its deficiencies.

## Figures and Tables

**Figure f1:**
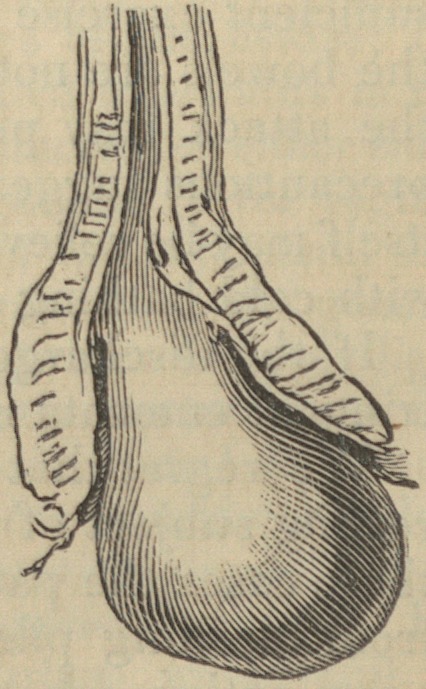


**Figure f2:**